# miR-146b-5p Plays a Critical Role in the Regulation of Autophagy in ∆per *Brucella melitensis*-Infected RAW264.7 Cells

**DOI:** 10.1155/2020/1953242

**Published:** 2020-01-19

**Authors:** Jiao Hanwei, Xin Nie, Huapei Zhu, Baobao Li, Feng Pang, Xiaohong Yang, Ruiyong Cao, Xiaojian Yang, Shu Zhu, Dongmei Peng, Yaying Li, Guohua Li, Zhenxing Zhang, Haifeng Huang, Kailian Xu, Tianjing Zhao, Ying Cheng, Chuangfu Chen, Li Du, Fengyang Wang

**Affiliations:** ^1^1 Hainan Key Lab of Tropical Animal Reproduction and Breeding and Epidemic Disease Research, College of Animal Science and Technology, Hainan University, Haikou, China; ^2^2 College of Animal Science, Southwest University, Chongqing, China; ^3^College of Animal Science and Technology, Shihezi University, Shihezi, China

## Abstract

*Brucella*-caused brucellosis is one of the most widespread worldwide zoonoses. Lipopolysaccharide (LPS) of *Brucella*, which functions as pathogen-associated molecular patterns (PAMPs), is an important virulence factor that elicits protective antibodies. *Per* of *B*. *melitensis* is involved in the biosynthesis of the O-side chain of LPS. Autophagy is a crucial element of the innate immune response against intracellular pathogens including *Brucella*. In this study, we observed that autophagy was inhibited in RAW264.7 cells infected with *Brucella melitensis* ∆per. And, a high-throughput array-based screen and qRT-PCR validation were performed to identify the differentially expressed miRNAs in RAW264.7 cells infected with *B. melitensis* M5-90 ∆per. The results suggested that *mmu-miR-146a-5p*, *mmu-miR-155-5p*, *mmu-miR-146b-5p*, and *mmu-miR-3473a* were upregulated and *mmu-miR-30c-5p* was downregulated. During *B. melitensis* M5-90 ∆per infection, the increased expression of *miR-146b-5p* inhibited the autophagy activation in RAW264.7 cells. Using a bioinformatics approach, *Tbc1d14* was predicted to be a potential target of *miR-146b-5p*. The results of a luciferase reporter assay indicated that *miR-146b-5p* directly targeted the 3′-UTR of *Tbc1d14*, and the interaction between *miR-146b-5p* and the 3′-UTR of *Tbc1d14* was sequence-specific. High-throughput RNA-Seq-based screening was performed to identify differentially expressed genes in Tbc1d14-expressing RAW264.7 cells, and these were validated by qRT-PCR. Among the differentially expressed genes, four autophagy associated genes, *IFNγ-inducible p47 GTPase* 1 (*IIGP1*), *nuclear receptor binding protein 2* (*Nrbp2*), *transformation related protein 53 inducible nuclear protein 1* (*Trp53inp1*), and *immunity-related GTPase family M member 1* (*Irgm1*), were obtained. Our findings provide important insights into the functional mechanism of LPS of *B. melitensis*.

## 1. Introduction

Brucellosis is one of the most widespread worldwide zoonoses, with 500,000 new cases reported each year, and is a serious public health problem [1]. Brucellosis is caused by *Brucella* spp., primarily *B. melitensis* and *B. abortus*. As the pathogen of brucellosis, *B. melitensis* stimulates a robust inflammatory response by LPS which bears a pathogen-associated molecular pattern (PAMP). When LPS binds to CD14, it transfers LPS to the TLR4/MD-2 complex, which triggers proinflammatory cytokine production [2–7].

LPS consists of lipid A, core oligosaccharide, and O-side chain [8]. Seven genes of *B. melitensis* are involved in the biosynthesis of the O-side chain—*wbkA*, *gmd*, *per*, *wzm*, *wzt*, *wbkB*, and *wbkC*—that encodes mannosyltransferase, GDP-mannose 4,6 dehydratase, perosamine synthetase (per), ABC-type transporter (integral membrane protein), ABC-type transporter (ATPase domain), a hypothetical protein of unknown function, and a putative formyl transferase, respectively. During biosynthesis of the O-side chain, perosamine is converted to GDP-perosamine by per. GDP-perosamine is polymerized into the O-side chain, translocated to the periplasm, transferred to the lipid A, and exported to the cell surface. The deletion of *Per* prevents O-side chain production [9].

Autophagy is an evolutionarily conserved catabolic process for the autophagosome-lysosomal degradation of bulk cytoplasmic contents in eukaryotes and can be activated by starvation and other physiological processes including bacterial infection [10]. Autophagy has different functional roles depending upon the species of pathogenic bacteria. For example, *Legionella pneumophila* [11] and *Coxiella burnetii* [12] are destroyed by autophagy, while other pathogens, including *Listeria monocytogenes* [13], *Mycobacterium tuberculosis* [14], and *Shigella* [15], have evolved multiple mechanisms to evade autophagy, leading to persistent infection and pathogenesis. To date, our understanding of autophagy is limited. Guo et al. observed that *B. melitensis* infection induced autophagy and that it favored the replication of *B. melitensis* in RAW264.7 [16]. Hamer et al. found that the Atg5-dependent autophagy pathway was dispensable for *Brucella* replication in mouse embryonic fibroblasts (MEFs) [17].

MicroRNAs (miRNAs) are about 20nt-long noncoding RNAs that posttranscriptionally regulate the gene expression [18]. Several miRNAs are associated with autophagic flux during bacterial infection. *miR-125a* inhibits autophagy activation and antimicrobial responses during mycobacterium infection [19]. *miR-155* promotes autophagy to eliminate intracellular mycobacterium by targeting *Rheb* [20]. *miR-4458*, *miR-4667*, and *miR-4668-5p* regulate the autophagy-associated elimination of *B. pseudomallei* by targeting ATG10 [21]. miR-146b inhibited autophagy in prostate cancer by targeting the PTEN/Akt/mTOR signaling pathway, and it may be a potential target for prostate cancer [22]. Studies have shown that miR-146b can target the signal pathway of NF-kB or mTOR, regulate the release of inflammatory factors, and then mediate the autophagy of intestinal cells [23]. However, the specific role of miRNAs in the regulation of autophagy during *Brucella* infection is largely unknown.

*Brucella* dysregulates monocyte and macrophage polarization through LC3-dependent autophagy [24]. *Brucella abortus* can activate the autophagy pathway by promoting a fibrotic phenotype in hepatic stellate cells [25]. Upon LPS + ATP stimulation, IL-1*β* was incorporated to an autophagic compartment, which indicated that an unconventional autophagy-mediated secretory pathway mediates IL-1*β* secretion in human neutrophils [26].

To elucidate the relationship between autophagy activation and *Brucella* infection, especially the functional role of miRNAs in the autophagy pathway during *Per* mutant *B. melitensis* infection, a specific research strategy was designed and performed (Supplementary [Supplementary-material supplementary-material-1]). miRNA gene profiling of macrophages infected with Per mutant *B. melitensis* was performed, and differentially expressed miRNAs were determined. Among the deregulated miRNAs, inhibitory effects of *miR-146b-5p* on autophagy were observed and the mechanism was analyzed.

## 2. Materials and Methods

### 2.1. Cells and Bacteria

RAW264.7 macrophages were grown as previously described [7]. *B. melitensis* vaccine strain M5-90, which still keeps residual virulence and may result in pregnant sheep abortion, provided by Dr. Hui Zhang of Shihezi University, was grown as previously described [7, 27].

### 2.2. Construction and Characterization of the per Deletion Mutant of *B. melitensis*

As shown in Supplementary [Supplementary-material supplementary-material-1], the genome of *B. melitensis* M5-90 was used as a template, and three pairs of primers, per-C-F and per-C-R, per-N-F and per-N-R, and Kana-F and Kana-R, were designed to amplify the upstream homologous sequence fragment of per, the downstream homologous sequence fragment of per, and kanamycin-resistance gene, respectively (Supplementary [Supplementary-material supplementary-material-1]). The three fragments were ligated into pMD20-T, respectively, and the recombinant plasmids were named pMD20-per-C, pMD20-per-N, and pMD20-Kana, respectively. pMD20-per-C was digested with SmaI and SacI, and the upstream homologous sequence fragment (per-C) was ligated into pGEM-7Zf (+), digested with the same enzymes, and the recombinant plasmid was named pGEM-C. pMD20-Kana was digested with XhoI and SmaI, and the fragment (Kanar) was ligated into pGEM-C, which was digested with the same enzymes, and the recombinant plasmid was named pGEM-C-K. pMD20-per-N was digested with ApaI and XhoI, the fragment (per-N) was ligated into pGEM-C-K, which was digested with the same enzymes, and the recombinant plasmid was named pGEM-C-K-N. Then, pGEM-C-K-N was electroporated into *B. melitensis* M5-90 competent cells, kanamycin-resistant colonies were selected, and recombination events were confirmed by PCR using Kanar-F and Kanar-R. The resulting strain was designated *B. melitensis* ∆per, which can grow in liquid medium (50 *μ*g/mL kanamycin). All constructs were confirmed by sequencing.

### 2.3. miRNA Array Profiling and Analysis (GEO: GSE126498)

RNA preparation and quantitation were performed as previously described [7]. Small RNA was extracted from RAW264.7-M5 and RAW264.7-M5-∆per, at an equal amount compared with RAW264.7 cells infected with *B. melitensis* M5-90 and *B. melitensis* M5-90 ∆per for 4 h, multiplicity of infection (MOI) is 10 : 1, a mirVanaTM microRNA Isolation kit (Ambion, Life technologies, USA) was used to extract the miRNA as described previously [7, 26]. miRNA microarray analyses were performed by LC-Bio Co (Hangzhou, China) as described previously [28].

### 2.4. mRNA Array Profiling and Analysis (GEO: GSE126343)

As previously described, PureLinkTM RNA Mini kit (Invitrogen, 12183018A) was used to extract high-quality total RNA from RAW264.7-M5 and RAW264.7-M5-∆per, respectively [7]. The RNA 6000 Nano LabChip kit (Agilent, 5067-1511) was used to determine the quality of RNA based on the Agilent Mus musculus Genome 4 × 44 K platform (V2, G4846A/26655) (LC-Bio Co., Hangzhou, China), and the Agilent 2100 Bioanalyzer was used for gene expression profiling.

### 2.5. qRT-PCR Validation for miRNAs and mRNAs

To determine miRNA expression levels, small RNA was extracted and qRT-PCR was performed to validate the differentially expressed miRNAs, miRNA specific primers, and U6 snRNA as internal control as previously described [28].

To determine mRNA levels, high-quality total RNA was extracted and qRT-PCR was performed to validate the differentially expressed mRNAs using the specific primers (Supplementary [Supplementary-material supplementary-material-1]) as previously described [7].

### 2.6. mmu-miR-146b-5p Target Binding tbc1d14: miRNA Transfection

mmu-miR-146b-5p mirVana® miRNA mimic (catalog number, MC10105), mmu-miR-146a-5p mirVana® miRNA mimic (catalog number, MC10722), and the scrambled control mirVana™ miRNA Mimic, Negative Control #1 (catalog number, 4464058), were purchased from Ambion (Austin, TX, USA). As previously described, the miRNA transfection experiments were performed using X-tremegene siRNA transfection reagent (Roche, 04476093001) [28].

### 2.7. Target Gene Prediction

The bioinformatics software, TargetScan, PicTar, and miRanda, were used to predict potential target genes of the confirmed differentially expressed miRNAs. After three group predicted genes were obtained, the overlap genes, predicted by all three algorithms, were selected for further ontology classification.

### 2.8. 3ʹ-Untranslated Region (3ʹ-UTR) Cloning, Mutation, and Luciferase Assay

Specific primers were designed (Supplementary [Supplementary-material supplementary-material-1]) to amplify the 3ʹ-UTR sequence of Tbc1d14 and 3ʹ-UTR of GAPDH from mouse cDNAs by PCR. Specific primers were designed (Supplementary [Supplementary-material supplementary-material-1]), and the putative miRNA-binding site 5ʹ-AGTTCTC-3ʹ (1937–1943) in the 3ʹ-UTR sequence of Tbc1d14 was mutated to 5ʹ-CTGGAGT-3ʹ by PCR. The constructs were validated by sequencing.

As previously described, RAW264.7 cells were seeded into a 96-well plate. After 24 h, the cells were cotransfected with the reporter plasmid and miR-146b-5p mimic or miR-NC mimic. At 36 h after transfection, the luciferase activity was determined [28].

### 2.9. Analysis of the Effect of mmu-miRNA-146b-5p on *B. melitensi*s-Mediated Autophagy of RAW264.7 cells: Western Blot Analysis

Western blot was performed as described previously [7]. The primary antibodies were mouse monoclonal MAP LC3*β* (G-9) (1 : 200 dilution) (Santa Cruz Biotechnology, sc-376404) and glyceradehyde-3-phosphate dehydrogenase (GAPDH) rabbit monoclonal Ab (Cell Signaling Technology, #5174), respectively. The secondary antibodies were horseradish peroxidase- (HRP-) labeled goat anti-mouse IgG (1 : 5000 dilution) (Santa Cruz Biotechnology, sc-2005) and HRP-labeled goat anti-rabbit IgG (1 : 5000 dilution) (Santa Cruz Biotechnology, sc-2004). BandScan software was used to quantify the protein expression.

### 2.10. Transmission Electron Microscopy

For transmission electron microscopy, RAW264.7 cells were infected with *B. melitensis* M5-90 and *B. melitensis* M5-90 ∆per for 4 h as previously described [7]. The cells were washed two times with 0.2 M sodium cacodylate buffer. Then, the cells were fixed with 4°C precooled 2.5% glutaraldehyde solution in phosphate buffer saline (PBS) for 30 min at 4°C. The fixed cells were postfixed in 1% OsO4, stained with 3% aqueous uranyl acetate, dehydrated in graded series of ethanol, and embedded in epoxy resin. Samples were then sectioned, stained with 2% uranyl acetate followed by 0.2% lead citrate, and examined on a JEM-1230 transmission electron microscope (JEOL, Japan).

### 2.11. Evaluation of the Role of tbc1d14 in *B. melitensis*-Mediated Autophagy of RAW264.7 Cells: Overexpression of tbc1d14 in RAW264.7

As previously described, the recombinant adenovirus expressing Tbc1d14 and rAdGFP-Tbc1d14, used in the current study, was prepared and infection experiments were performed [28]. Briefly, RAW264.7 was seeded in a 12-well plate at a concentration of 2 × 105 cells per well (coverslips were placed on the bottom of the plate). The cells were infected with rAdGFP-Tbc1d14 at ∼80% confluency. After 12 h, the infection was performed with a multiplicity of infection (MOI) of 100 plaque-forming units/cell in 1 ml infection buffer at room temperature. After 18 h, the medium was changed with the fresh medium, and after 48 h, the cells were washed with phosphate-buffered saline (PBS) and were harvested for further assays.

### 2.12. Transcriptome Experiment and Analysis

As previously described, an mRNA library was constructed and sequencing was performed with an Illumina 2000/2500 sequence platform (LC Sciences, USA). Clean reads of 36 nt in length were obtained by removing adaptor sequences, tags with low-quality sequences, and unknown nucleotides N (*N* > 2). The obtained valid data were aligned to a database (ftp://ftp.ensembl.org/pub/release-77/fasta/musmusculus/) using Bowtie software. Reads per kilobase of exon model per million mapped reads (RPKM) values were used to normalize the number of fragments. Based on the expression levels, the differentially expressed genes (DEGs), their corresponding attributes, fold changes (in log2 scale), and *p* values were obtained, and DEGs with a *p* value ≤0.05 and |log2 fold-change| (|log2FC|) ≥1 were identified.

As previously described, GO was conducted for the functional classification of DGEs and pathway analysis was carried out using KEGG [28].

### 2.13. Statistical Analysis

Student's *t* test and one-way ANOVA were used to analyze the statistical significance of the differences between mean values for the various experimental groups and controls. Data are expressed as the mean ± standard deviation (SD) from triplicate experiments. A *p* value less than 0.05 was considered statistically significant.

## 3. Results

### 3.1. Autophagy Is Inhibited in RAW264.7 Cells Infected with *B. melitensis* ∆per

**Figure 1:**
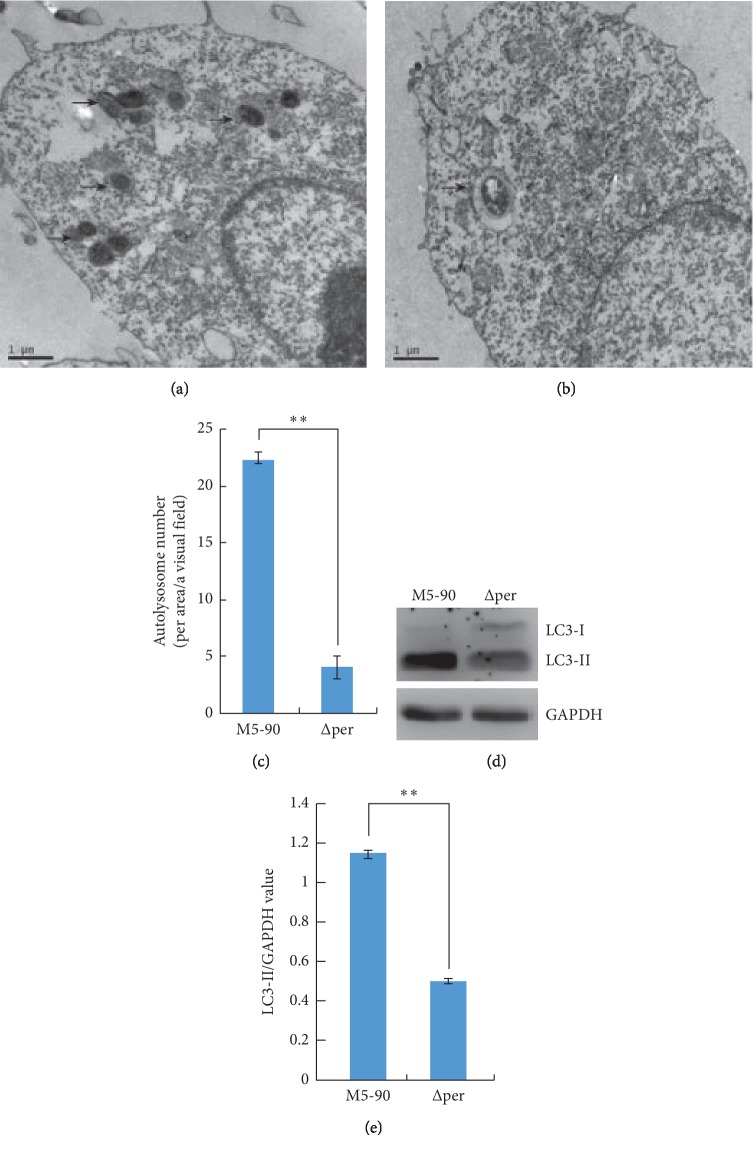
Autophagy is inhibited in RAW264.7 cells infected with *B. melitensis* ∆per. (a, b) Transmission electron microscopy examination of RAW264.7 infected with *B. melitensis* M5-90 or *B.melitensis* ∆per for 4 h, respectively. Arrow indicates autolysosome. (c) The number of autolysosome of RAW264.7 infected with *B. melitensis* ∆per for 4 h was significantly decreased. (d) The levels of LC3-II/GAPDH. RAW264.7 cells were infected with *B. melitensis* M5-90 or *B. melitensis* ∆per for 4 h, respectively, and then the total cell lysates were prepared and subjected to immunoblot analysis using monoclonal anti-LC3-I antibody and polyclonal antibody against LC3-II. (e) The quantification of LC3-II/GAPDH levels in *d* with BandScan5.0 (*n* = 3). Scale bars are 1 *μ*m. Data are mean ± SD from three independent experiments. ^*∗*^*p* < 0.05; ^*∗∗*^*p* < 0.01.

Autophagy is the end result of a complex signaling pathway that leads to the generation of a double-membrane organelle, the autophagosome. Autophagosomes are generated at the phagophore assembly site (PAS). The autophagosomes fuse with lysosomes to generate autolysosomes, within which the autophagosomal inner membrane and cargo are degraded [29]. Transmission electron microscopy (TEM) is used to visualize double-membrane organelles and has become the “gold standard” for autophagy confirmation. In this study, *B. melitensis* ∆per was constructed (Supplementary [Supplementary-material supplementary-material-1]), and RAW264.7 cells were infected for 4 h with *B. melitensis* M5-90 and *B. melitensis* ∆per, respectively. Samples from RAW264.7 infected with *B. melitensis* M5-90 were named the M group, and thirty images were obtained. Samples from RAW264.7 infected with *B. melitensis* M5-90-∆per were named the P group, and thirty images were also obtained. The numbers of autophagosomes and autolysosomes in the M and P groups were calculated. Autolysosome numbers in the P group were significantly decreased compared with those in the M group (negative control) (Figures 1(a)–1(c)).

Autophagy marker, light chain 3 (LC3), was originally identified as a subunit of microtubule-associated proteins 1A and 1B (MAP1LC3). Cleavage of LC3 at the carboxy terminus immediately following synthesis yields a cytosolic protein, LC3-I. During autophagy, LC3-I is converted to LC3-II through lipidation by an ubiquitin-like system involving Atg7 and Atg3. The LC3 moiety is conjugated to phosphatidylethanolamine (PE) on autophagosomal precursor membranes. The presence of LC3 in autophagosomes and the conversion of LC3-I to the lower migrating form LC3-II have been used as indicators of autophagy [30]. In this study, we evaluated the autophagy activation in RAW264.7 infected with M5-90 or ∆per, and western blot assay was used to analyze the processing of LC3 (conversion from LC3-I to LC3-II). The levels of LC3-II/GAPDH in RAW264.7 cells infected with *B. melitensis* M5-90-∆per was significantly lower than that in RAW264.7 cells infected with *B. melitensis* M5-90 (Figures 1(d) and 1(e)).

Taken together, we observed the autophagy activation was inhibited in RAW264.7 cells infected with *B. melitensis* ∆per.

### 3.2. *B. melitensis* ∆per Induces Differential miRNA Levels in RAW264.7

**Figure 2:**
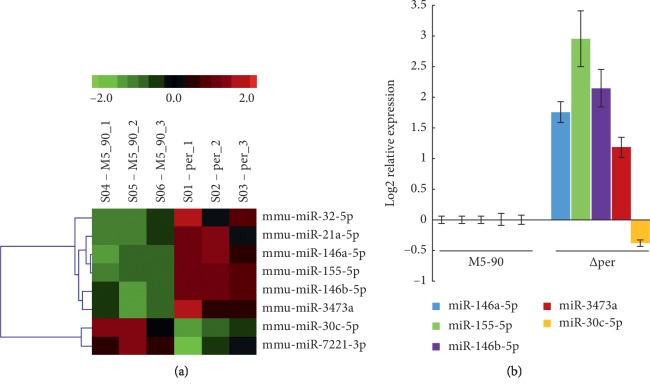
The differentially expressed miRNA obtained from RAW264.7-M5 and RAW264.7-∆per. (a) Heat-map indicated the upregulation of mmu-miR-32-5p, mmu-miR-21a-5p, mmu-miR-146a-5p, mmu-miR-155-5p, mmu-miR-146b-5p, and mmu-miR-3473a (red) and the downregulation of mmu-miR-30c-5p and mmu-miR-7221-3p (green) (*p* < 0.05). (b) Relative expression levels of mmu-miR-146a, mmu-miR-155-5p, mmu-miR-146b-5p, mmu-miR-3473a, and mmu-miR-30c-5p were confirmed by qRT-PCR. Data are mean ± SD from three independent experiments. ^*∗*^*p* < 0.05; ^*∗∗*^*p* < 0.01.

The results of the array indicated that there were eight differentially expressed miRNAs in RAW264.7 infected with *B. melitensis* M5-90 ∆per (*p* < 0.01;, they were mmu-miR-32-5p, mmu-miR-21a-5p, mmu-miR-146a-5p, mmu-miR-155-5p, mmu-miR-146b-5p, mmu-miR-3473a, mmu-miR-30c-5p, and mmu-miR-7221-3p (Figure 2(a); Table 1). After the qRT-PCR validation assay was performed, the upregulation of mmu-miR-146a-5p, mmu-miR-155-5p, mmu-miR-146b-5p, and mmu-miR-3473a and the downregulation of mmu-miR-30c-5p was confirmed (Figure 2(b)). However, the qRT-PCR results of mmu-miR-32-5p, mmu-miR-21a-5p, and mmu-miR-7221-3p were not consistent with that of array-based screening (Supplementary [Supplementary-material supplementary-material-1]).

### 3.3. *B. melitensis* ∆per Induces Differential mRNA Levels in RAW264.7

**Figure 3:**
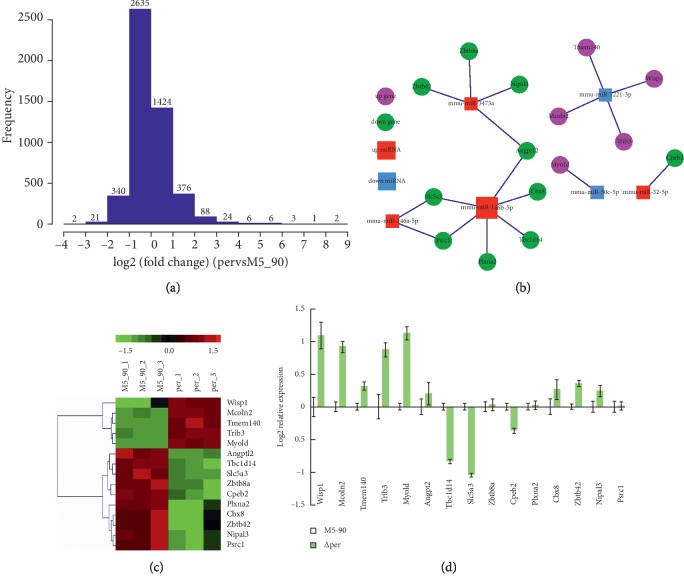
The differentially expressed mRNAs obtained from RAW264.7-M5 and RAW264.7-∆per by the array-based initial screen. (a) The fold change between the biological samples. The *X*-axis indicated log2 (FC), while the *Y*-axis indicated the numbers of the differentially expressed gene transcripts. (b) miRNA-mRNA interaction network of differentially expressed miRNAs and their fifteen putative targets. mmu-miR-146a, mmu-miR-155-5p, and mmu-miR-146b-5p were upregulated (red), and their ten putative target genes were downregulated (green); mmu-miR-3473a and mmu-miR-30c-5p were downregulated (blue), and their five putative target genes were upregulated (pink). (c) Heat-map obtained from mRNA array indicated the upregulation of five genes (Wisp1, Mcoln2, Tmem140, Trib3, and Myold) (red) and the downregulation of ten genes (Angptl2, Tbc1d14, Slc5a3, Zbtb8a, Cpeb2, Plxna2, Cbx8, Zbtb42, Nipal3, and Psrc1) (green) (*p* < 0.05). (d) Relative expression levels of the fifteen targets gene were validated by qRT-PCR. nTbc1d14 and Slc5a3 were identified as the differentially expressed genes by qRT-PCR. Data are mean ± SD from three independent experiments. ^*∗*^*p* < 0.05; ^*∗∗*^*p* < 0.01.

To obtain the potential targets of these miRNAs in RAW264.7 infected with B. melitensis M5-90 ∆per, a high-throughput array-based screen was performed. The results indicated that the number of dysregulated genes (fold change >2) in RAW264.7 infected with B. melitensis ∆per was 869 (*p* < 0.05) (Figure 3(a)). mRNA microarray and extensive bioinformatics analysis (TargetScan, miRanda, and PicTar) suggested that, among 869 differentially expressed genes, 15 genes were predicted to be potential targets of the five confirmed miRNAs, mmu-miR-146a-5p, mmu-miR-155-5p, mmu-miR-146b-5p, mmu-miR-3473a, and mmu-miR-30c-5p (Figures 3(b) and 3(c); Table 2).

To validate the corresponding relation between miRNA-potential target genes, specific primers were designed (Supplementary [Supplementary-material supplementary-material-1]) and qRT-PCR was performed. The results indicated that the downregulation of *Slc5a3* (*solute carrier family 5* (*inositol transporters*), *member 3*) and *Tbc1d14* (*TBC1 domain family, member 14*) was confirmed (Figure 3(d)) and suggest that the corresponding relation, *miR-146a-5p-Slc5a3, miR-146b-5p-Slc5a3*, and *miR-146b-5p-Tbc1d14*, was reasonable.

To examine whether *miR-146a-5p* regulates Slc5a3 expression levels, a mmu-miR-146a-5p mirVana® miRNA mimic was transfected into cells expressing endogenous Slc5a3. The scrambled mirVana™ miRNA mimic was used as negative control. The results of qRT-PCR assay indicated that there were no obvious changes in Slc5a3 expression levels in cell lines transfected with *miR-146a-5p* (Supplementary [Supplementary-material supplementary-material-1]). In the same way, qRT-PCR assay was performed to indicate that there were no obvious changes in Slc5a3 protein levels in cell lines transfected with *miR-146b-5p* mirVana® miRNA mimic (Supplementary [Supplementary-material supplementary-material-1]).

**Figure 4:**
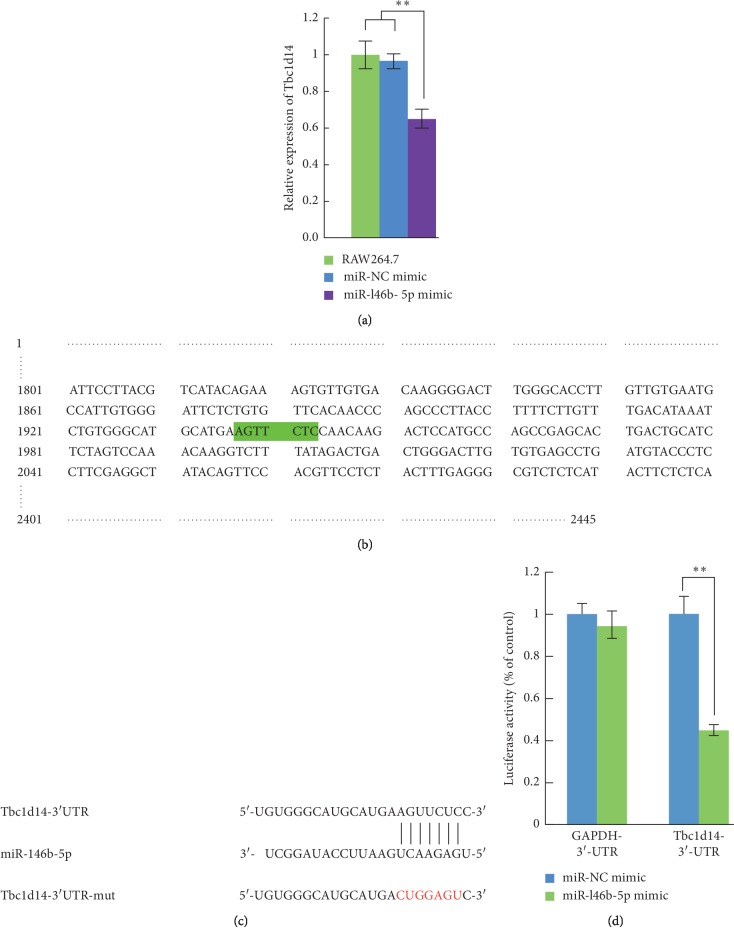
mmu-miR-146b-5p targets Tbc1d14. (a) Tbc1d14 mRNA levels in cells transfected with miR-NC mimic and miR-146b mimic. RAW264.7 were transfected with miR-146b mimic (100 nM), and the Tbc1d14 mRNA levels were measured at 24 h after transfection by qRT-PCR assays. Control (con) cells were transfected with miR-NC mimic. (b, c) Mutation of Tbc1d14 3′UTR. The binding sites of miR-146b in the Tbc1d14 3′UTR (AGUUCUCC) were predicted by bioinformatics software. AGUUCUC (the binding sites) were mutated into CUGGAGU (red). (d) The miR-146b-5p binding sites in mouse Tbc1d14-3′UTR mediates the repression of luciferase activities in cells. Data are mean ± SD from three independent experiments. ^*∗*^*p* < 0.05; ^*∗∗*^*p* < 0.01.

To examine whether *miR-146b-5p* regulates Tbc1d14 expression levels, a mmu-miR-146b-5p mirVana® miRNA mimic was transfected into cells expressing endogenous Tbc1d14. The scrambled mirVana™ miRNA mimic was used as negative control. The results of qRT-PCR assay indicated that the expression of Tbc1d14 was downregulated in RAW264.7 cells transfected with the mmu-miR-146b-5p mimic (Figure 4(a)). These results demonstrated that *mmu-miR-146b-5p* negatively regulates Tbc1d14 expression. Therefore, *miR-146b-5p* and Tbc1d14 were selected for further study.

### 3.4. miR-146b-5p Targets tbc1d14

Identification of the functional target sequences of miR-146b-5p is necessary to determine the regulatory effects of this miRNA on autophagy activation. To confirm the in silico prediction and show that Tbc1d14 is the target of miR-146b-5p, a luciferase reporter assay was performed. The 3ʹ-UTR fragment of mouse Tbc1d14 (NM-001113362.1) and mouse GAPDH (NM 001289726) were cloned, and then cloned into a pmirGLO-luciferase reporter plasmid to generate pmirGLO-luciferase-Tbc1d14 -3ʹ-UTR-WT and pmirGLO-luciferase-GAPDH-3ʹ-UTR-WT. The mutation of 3ʹ-UTR fragment of mouse Tbc1d14 was cloned, and then cloned into pmirGLO-luciferase reporter plasmid to generate pmirGLO-luciferase-Tbc1d14-3ʹ-UTR-mut (Figure 4(b)). RAW264.7 cells were co-transfected with the wild-type GAPDH-3ʹ-UTR reporter plasmid and miR-146b-5p mimic or miR-NC mimic, with the wild-type Tbc1d14 3ʹ-UTR reporter plasmid and miR-146b-5p mimic or miR-NC mimic, and with the mutated Tbc1d14-3ʹ-UTR reporter plasmid and miR-146b-5p mimic or miR-NC mimic. The luciferase activity experimental results suggested that compared with that of co-transfection with pmirGLO-luciferase-Tbc1d14-3ʹ-UTR-WT and miR-NC mimics, the luciferase activity in RAW264.7 cells co-transfected with pmirGLO-luciferase Tbc1d14-3ʹ-UTR-WT and miR-146b-5p mimic was reduced by about 60%. Furthermore, the mutation of the miR-146b-5p binding site abrogated the inhibitory effects of luciferase activity, thereby indicating miR-146b-5p reduced luciferase activity via Tbc1d14-3ʹ-UTR (Figures 4(c) and 4(d)).

### 3.5. miR-146b-5p Inhibits Autophagy Activation in RAW264.7 Cells during *B. melitensis* Infection

**Figure 5:**
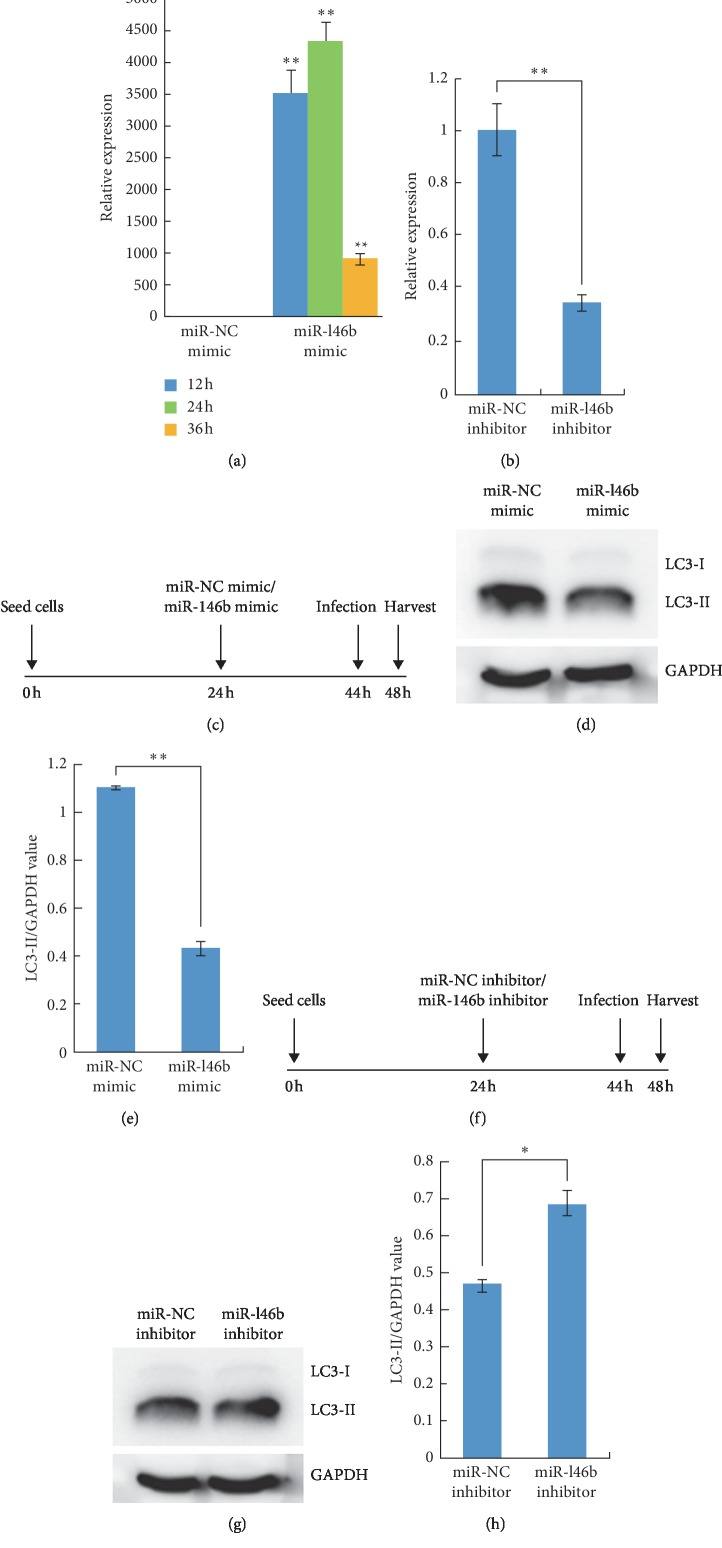
mmu-miR-146b-5p inhibits the autophagy activation in RAW264.7 cells during *B. melitensis* infection. (a) mmu-miR-146b-5p expression levels in RAW264.7 cells transfected with miR-146b-5p mimic were examined by qRT-PCR at 12 h, 24 h, and 36 h after transfection. (b) mmu-miR-146b-5p expression levels in RAW264.7 cells transfected with miR-146b-5p inhibitor were examined by qRT-PCR at 24 h after transfection, and the expression level of miR-146b-5p was downregulated significantly by the miR-146b-5p inhibitor. (c) RAW264.7 cells were seeded at 0 h, were transfected with miR-NC mimic or miR-146b mimic after 24 h, and were infected with *B. melitensis* M5-90 after 44 h, and the total protein was extracted after 48 h. (d) Total cell lysates in C were then prepared and subjected to immunoblot analysis using monoclonal anti-LC3-I antibody and polyclonal antibody against LC3-II. (e) The quantification of LC3-II/GAPDH levels in *D* with BandScan5.0 (*n* = 3). (f) RAW264.7 cells were seeded at 0 h, were transfected with miR-NC inhibitor or miR-146b inhibitor after 24 h, and were infected with B. melitensis M5-90 after 44 h, and the total protein was extracted after 48 h. (g) Total cell lysates in F were then prepared and subjected to immunoblot analysis using monoclonal anti-LC3-I antibody and polyclonal antibody against LC3-II. (h) The quantification of LC3-II/GAPDH levels in *G* with BandScan5.0 (*n* = 3). Data are mean ± SD from three independent experiments. ^*∗*^*p* < 0.05; ^*∗∗*^*p* < 0.01.

To investigate the role of miR-146b-5p in autophagy activation in RAW264.7 cells during *B. melitensis* infection, RAW264.7 cells were transfected with miR-NC mimic or miR-146b-5p mimic at 24 h after seeding and infected with *B. melitensis* M5-90 at 44 h after seeding and the protein was extracted after infection for 4 h (Figure 5(c)). The western blotting results showed that the levels of LC3-II/GAPDH in miR-146b-5p mimic transfected group were significantly lower than those of the miR-NC mimic transfected group (Figures 5(d) and 5(e)). We evaluated the effects of miR-146b-5p inhibitor in RAW264.7 cells during *B. melitensis* infection. RAW264.7 cells were transfected with miR-NC inhibitor or miR-146b-5p inhibitor, and qRT-PCR was performed to determine the expression level of miR-146b-5p. The results indicated that, at 24 h after transfection, the expression level of miR-146b-5p was downregulated significantly, indicating that the miR-146b-5p inhibitor inhibited the expression of miR-146b-5p (Figure 5(b)). We performed western blotting assay in the same way, and the results indicated that the levels of LC3-II/GAPDH in the miR-146b-5p inhibitor transfected group was significantly higher than those of the miR-NC inhibitor infected group (Figures 5(f)–5(h)). Taken together, these data demonstrated that the forced expression of miR-146b-5p inhibited the autophagy activation in RAW264.7 cells during *B. melitensis* infection.

### 3.6. Tbc1d14 Modulates miR-146b-5p-Mediated Autophagy Activation in RAW264.7 Cells during *B. melitensis* Infection

**Figure 6:**
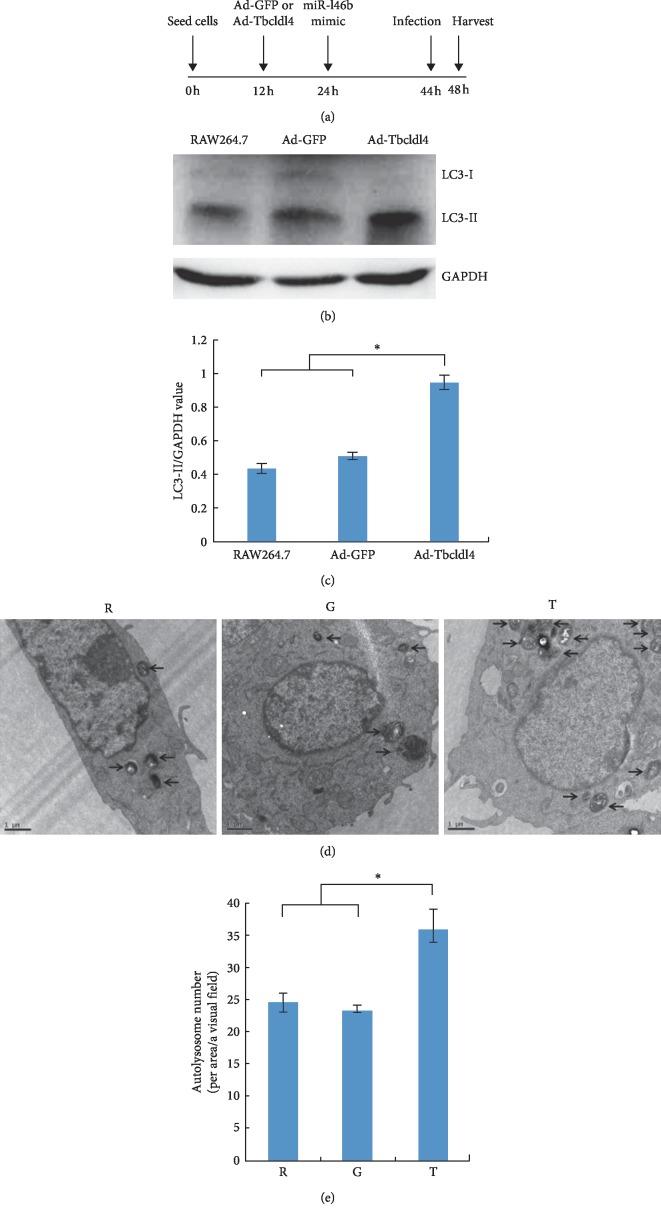
Tbc1d14 contributes to miR-146b-5p-mediated inhibition of autophagy during *B. melitensis* infection. (a) RAW264.7 cells were seeded at 0 h, were infected with Ad-EGFP or Ad-Tbc1d14 after 12 h, were transfected with miR-146b mimic after 24 h, were infected with *B. melitensis* M5-90 after 44 h, and the total protein was extracted after 48 h. (b) Western blot analysis of the LC3-I and LC3-II protein expression. (c) The quantification of LC3-II/GAPDH levels in C with BandScan5.0 software (*n* = 3). (d) Transmission electron microscopy observation of the autolysosome in Ad-Tbc1d14 infected RAW264.7 group (Group T), Ad-EGFP infected RAW264.7 group (Group G) as negative control, and RAW264.7 group as blank control (Group R). (e) Statistical analysis for the number of autolysosomes in *T*, *G*, and *R*. Scale bars are 1 *μ*m. Data are mean ± SD from three independent experiments. ^*∗*^*p* < 0.05; ^*∗∗*^*p* < 0.01.

Tbc1d14 overexpression by rAdGFP-Tbc1d14 was induced to elucidate the function of Tbc1d14 in autophagy activation in RAW264.7 cells during *B. melitensis* infection. RAW264.7 cells were infected with rAdGFP-Tbc1d14 at 12 h, transfected with miR-146b-5p at 24 h, and infected with B. melitensis at 44 h. Cell total protein was extracted for western blot analysis, and at same time, the cells were fixed and prepared for transmission electron microscopy (Figure 6(a)). The levels of LC3-II/GAPDH in Tbc1d14-expressing RAW264.7 cells were significantly higher than those in EGFP (only)-expressing RAW264.7 cells and those in RAW264.7 cells (Figures 6(b) and 6(c)). Transmission electron microscopy demonstrated that the number of autolysosomes in Tbc1d14-expressing RAW264.7 cells was significantly higher than that in EGFP (only)-expressing RAW264.7 cells and RAW264.7 cells (Figures 6(d) and 6(e)).

### 3.7. Overexpression of tbc1d14 in RAW264.7 Induces Four Differentially Expressed Autophagy-Associated Genes

After recombinant adenovirus infection, Tbc1d14-expressing RAW264.7 cells were obtained and designated T, and EGFP (only)-expressing RAW264.7 cells were obtained and designated E. RAW264.7 cells were used as a blank control and designated R. Using the Illumina paired-end RNA-seq approach, a cDNA library of T, E, and R was sequenced. The purified RNA was ligated with a preadenylated 3′ adapter, which enables the subsequent ligation of the 5′ adapter. Based on the adapter sequence, a reverse transcription followed by PCR was performed to create cDNA constructs. The mean insert size of the single-end libraries was 250 bp (±50 bp), and 113,889,072 single-end reads of with a length of 50 bp were acquired. The total read length of three samples was 5.71 gigabases (Gb). After removing the low-quality reads, a total of 5.669 Gb of valid data were produced. The Q20 was above 90%.

Alignment of the sequence reads against the reference genome yielded about 82,371,423 aligned reads across the three samples, for which the ratio of pair reads was about 70.9% and the ratio of unique map was about 66.48%. Multiposition-matched reads (<10%) were excluded from further analyses. The distribution of the density of the sequence was normal. These data satisfied the requirements of further analyses.

**Figure 7:**
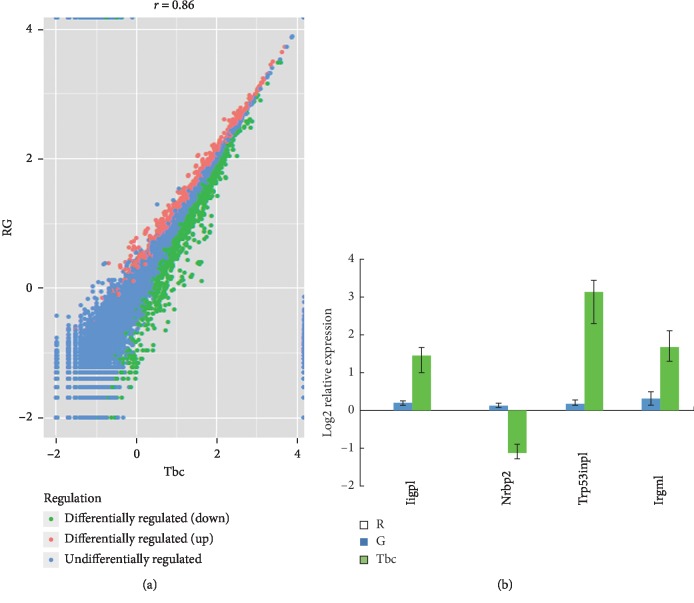
Overexpression of Tbc1d14 in RAW264.7 induced the expression dysregulation the autophagy-associated genes. (a) The scatter plot of the significant differential expressed genes of T vs RG. (b) qRT-PCR validation of the significantly differentially expressed autophagy-associated genes. Ad-Tbc1d14-infected RAW264.7 group (Group Tbc), Ad-EGFP-infected RAW264.7 group (Group G) as negative control, and RAW264.7 group as blank control (Group R). Data are mean ± SD from three independent experiments. ^*∗*^*p* < 0.05; ^*∗∗*^*p* < 0.01.

Compared with the expression values of E and R, the DEGs from T, with a *p* value ≤0.05 and |log2 fold-change |(|log2FC|) ≥1, showed a significant differential gene expression: genes whose log2FC was >1 were upregulated and genes whose log2FC was <1 are downregulated. GO functional enrichment analysis identified four autophagy-associated genes, *Iigp1*, *Nrbp2*, *Trp53inp1*, and *Irgm1*. *Iigp1*, *Irgm1*, and *Trp53inp1* were upregulated and *Nrbp2* was downregulated. To confirm the RNA-Seq data, specific primers were designed (Supplementary [Supplementary-material supplementary-material-1]), and qRT-PCR was performed using GAPDH as an internal control. The results confirmed that the mRNA levels of *IIGP1*, *Irgm1*, *and Trp53inp1* were upregulated; and the mRNA levels of *Nrbp2* were downregulated (Figure 7).

## 4. Discussion

Intracellular pathogens have adopted various strategies to evade host defense mechanisms including autophagy. For *Brucella*, little is known regarding its activity in modulating autophagy. Understanding the mechanisms of autophagy modulation in host response to *Brucella* infection will provide crucial information for the prevention and therapy of brucellosis. Host cells activate the killing function of the immune system against invading *Brucella* [31]. However, in order to survive, *Brucella* has evolved the function of escaping capture and killing. The formation of *Brucella*-containing vacuole (BCV) ensures the replication niche of intracellular *Brucella*. Autophagy initiation proteins such as BECLIN1, PI3K, ULK1, and Atg14L can affect the activation of autophagic BCV (aBCV), which influenced the survival viability of *Brucella* and destroyed the host cell membrane trafficking pathways [32, 33]. Here, we report the first study to show that autophagy activation in RAW264.7 cells infected with *B. melitensis* ∆per can be inhibited. *B. melitensis* ∆per upregulated *miR-146b-5p* which targets *Tbc1d14*, and Tbc1d14 modulates miR-146b-5p-mediated autophagy activation in RAW264.7 cells during *B. melitensis* infection.

*miR-146b*, a TLR-responsive miRNAs, plays an important functional role during inflammatory responses. LPS induces the expression of *miR-146b*, which targets multiple elements involved in the TLR4 signaling pathway, suggesting that *miR-146b* is a candidate feedback modulator of the LPS response. The target genes include *TLR4*, *myeloid differentiation primary response* (*MyD88*), *interleukin-1 receptor-associated kinase 1* (*IRAK-1*), and *TNF receptor-associated factor 6* (*TRAF6*) [34]. In human monocytes, the enforced expression of *miR-146b* downregulated the LPS-dependent production of IL-6, TNF-*α*, IL-8, CCL3, CCL2, CCL7, and CXCL10 [35]. Additionally, *miR-146b-5p* also regulated both apoptotic and antiapoptotic genes induced by EGF treatment [36].

Autophagy is an early defense response against intracellular pathogens and is characterized by the formation of autophagosomes. The exact molecular mechanism of autophagosome formation and the origin of the autophagosomal membrane is complex. Recent studies have identified many components that mediate this complicated cellular process. Eighteen ATG proteins including ATG1, ATG2A, ATG3, ATG4A-D, ATG5, ATG6, ATG7, ATG8, ATG9A, ATG10, ATG12, ATG13, ATG14L, ATG 16L1, ATG17, ATG18, and ATG101 and five additional factors including VPS34, p150, AMBRA1, VMP1, and DFCP1 are grouped in five functional complexes and compose the core autophagy machinery in high eukaryotes [37].

Our experiments suggest that *miR-146b-5p* targets *Tbc1d14* (*TBC1 domain family, member 14*). Tbc1d14 is a Tre-2/Bub2/Cdc16 (TBC) domain-containing protein that colocalizes and interacts with the autophagy kinase ULK1 [35]. Longatti and Tooze identified Tbc1d14 as a negative regulator of starvation-induced autophagy activation that controls the delivery of membranes from RAB11-positive recycling endosomes to forming autophagosomes [29]. Tbc1d14 binds to the TRAPP complex via an N-terminal 103 amino acid region, and the overexpression of this region inhibits autophagy activation. Lamb et al. proposed a model whereby TBC1D14 and TRAPPIII regulate a constitutive trafficking step from peripheral recycling endosomes to the early Golgi, maintaining the cycling pool of ATG9 required for the initiation of autophagy activation [38]. TBC1D14 influences Golgi and endosomal structure and function in other cell lines, although the effect on these organelles in the RAW264.7 cell line has not been clearly defined in this study. Interestingly, we observed that, under *B. melitensis* infection, Tbc1d14 overexpression elevated the *miR-146b-5p* mediating the autophagy activation in RAW264.7 cells. The different functional roles of Tbc1d14 during autophagy induction might be as follows: (1) it depends on the starvation that Longatti and Tooze identified Tbc1d14 as a negative regulator of autophagy activation; however, it is under *B. melitensis* infection where we observed that Tbc1d14 overexpression elevates the autophagy activation in RAW264.7 cells. (2) The cell line used was HEK293A in the study of Longatti and Tooze; however, we used RAW264.7 cells for the analysis of autophagy activation. It is obvious that the inhibition or stimulation of autophagy activation depends on different stimuli. We tried to use the mRFP-GFP-LC3 adenovirus fusion protein to track autophagy by using the confocal laser scanning microscope; the experiments were not interpretable due to the unusual phenotypes displayed by the cells.

We observed that the overexpression of Tbc1d14 in RAW264.7 induced the differential expression of four autophagy associated genes, *Iigp1*, *Irgm1*, *Trp53inp1*, and *Nrbp2*, which did not appear in transcriptome array profile (GEO: GSE126343). Atg5 is involved in the trafficking of degradative vesicles and MHC II to mycobacterial autophagosomes [39]. In autophagy-deficient Atg5-/- cells, GAS (group A *Streptococcus*) survived and multiplied and was released from the cells [40]. Under *Toxoplasma gondii* infection, Atg5 was required for the recruitment of *IFNγ-inducible p47 GTPase IIGP1* to the vacuole membrane, which is an important component of the cellular machinery that controls the bacteria [41]. Irgm1 belongs to a family of immunity-related GTPases that function in cell-autonomous resistance against intracellular pathogens in mice [42]. Irgm1 induced autophagy activation and generated large autolysosomal organelles as a mechanism to eliminate intracellular *Mycobacterium tuberculosis* [43]. Irgm1 regulated the survival of mature effector CD4(+) T lymphocytes by protecting them from IFN*γ*-induced autophagic cell death [44]. TP53INP1s are functionally associated with p73 to regulate cell cycle progression and apoptosis, independent from p53 [45]. NRBP2 is a 55–60 kDa protein mainly present in the cytoplasmic location [46]. In sertoli cells, TiO2 NPs caused severe testicular oxidative damage and/or apoptosis, excessive production of reactive oxygen species, and peroxidation of lipids, proteins, and DNA, and the antioxidant capacity was also reduced significantly. The exposure to TiO2 NPs resulted in the upregulation of Nrbp2 [47].

**Figure 8:**
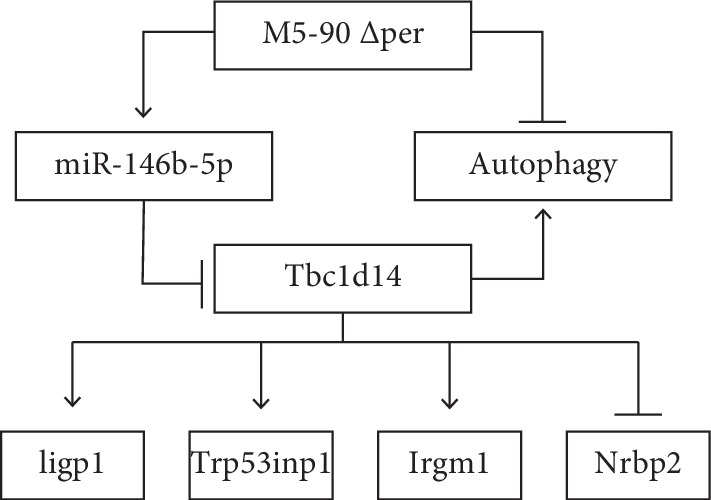
Model of mmu-miR-146b-5p involving in autophagy activation during Per mutant *B. melitensis* infection.

These data demonstrate that LPS plays an important role in *B. melitensis*-induced autophagy through modulating miR-146b-5p and its target TBC1D14, and four autophagy-related genes, *Iigp1*, *Nrbp2*, *Trp53inp1*, and *Irgm1*, involved in the process (Figure 8). Further work is required to determine how these four genes are involved in *B. melitensis*-induced autophagy activation.

## Figures and Tables

**Table 1:** The significant differential expressed miRNAs by microarray analysis.

Reporter name	*p* value	Group1 M5-90	Group2 Per	Log2 (*G*2/*G*1)	qRT-PCR ΔΔCт	FDR
		Mean	Mean			
mmu-miR-32-5p	3.88*E* − 02	88	111	0.34	0.74	0.0437
mmu-miR-21a-5p	1.91*E* − 02	3,465	6,380	0.88	0.72	0.0382
mmu-miR-146a-5p	7.11*E* − 03	877	2,352	1.42	1.76	0.02844
mmu-miR-155-5p	6.26*E* − 04	388	2,214	2.51	2.95	0.005008
mmu-miR-146b-5p	1.63*E* − 02	391	1,256	1.68	2.14	0.0382
mmu-miR-3473a	3.67*E* − 02	77	113	0.55	1.18	0.0437
mmu-miR-30c-5p	3.57*E* − 02	2,499	1,914	−0.39	0.38	0.0437
mmu-miR-7221-3p	4.37*E* − 02	48	27	−0.81	−0.38	0.0437

**Table 2:** The significantly differentially expressed targets of the validated significantly differentially expressed mRNAs by microarray analysis.

Gene symbol	*p* value	Group1M5-90	Group2Per	Log2 (*G*2/*G*1)	qRT-PCR ΔΔCт	FDR
		Mean	Mean			
Wisp1	4.43*E* − 02	25.97	95.87	1.88	−1.09	0.0443
Mcoln2	1.82*E* − 04	70.92	271.37	1.94	−0.92	0.001365
Tmem140	2.91*E* − 03	59.17	133.73	1.18	−0.32	0.006235714
Trib3	3.98*E* − 04	379.43	828.22	1.13	−0.87	0.0014925
Myo1d	7.52*E* − 06	1295.14	2623.70	1.02	−1.12	0.0001128
Angptl2	3.03*E* − 04	144.82	48.02	−1.95	−0.20	0.0014925
Tbc1d14	3.93*E* − 03	304.55	105.05	−1.54	0.84	0.00736875
Slc5a3	2.18*E* − 02	137.85	55.35	−1.32	0.99	0.025153846
Zbtb8a	1.12*E* − 03	48.60	12.51	−1.96	−0.03	0.00336
Cpeb2	9.34*E* − 03	51.16	23.99	−1.09	0.37	0.015566667
Plxna2	1.68*E* − 02	156.77	73.01	−1.10	−0.02	0.025153846
Cbx8	2.17*E* − 02	89.53	28.27	−1.66	−0.26	0.025153846
Zbtb42	3.32*E* − 02	29.15	12.60	−1.21	−0.36	0.035571429
Nipal3	1.45*E* − 03	117.88	47.41	−1.31	−0.24	0.003625
Psrc1	1.92*E* − 02	149.46	74.07	−1.01	−0.01	0.025153846

## Data Availability

The miRNA array data and the agilent mRNA array data were deposited into Sequence Read Archive (SRA) of National Center of Biotechnology Information (NCBI) with the GEO number of GSE126498 and GSE126343.

## Supplementary Materials

Supplementary Materials Supplementary Figure 1: the technology roadmap: miRNA and mRNA profiling of macrophages infected with *B. melitensis* M5-90 ∆per was performed and differentially expressed miRNAs and mRNA were determined. Among the deregulated miRNA-mRNA networks, inhibitory effects of miR-146b-5p on autophagy were observed and the mechanism was analyzed. Supplementary Figure 2: construction and characterization of the per deletion mutant of *B. melitensis* M5-90: Kana gene fragment replaced the coding region of per gene in *B. melitensis* M5-90 using homologous recombination to construct deleted strains of *B. melitensis* M5-90 ∆per. Supplementary Figure 3: validation for the relative expression of Slc5a3 of RAW264.7 cell transfected with miR-146a-5p mimic and miR-NC mimic by qRT-PCR. Supplementary Figure 4: validation for the relative expression of Slc5a3 of RAW264.7 cell transfected with miR-146b-5p mimic and miR-NC mimic by qRT-PCR. STABLE 1: the primers designed for constructing the Per deletion mutant of *B. melitensis*. STABLE 2: the primers designed for qRT-PCR validation of target gene. STABLE 3: the primers designed for cloning of Tbc1d14 3′-UTR and GAPDH3′-UTR. STABLE 4: the primers designed for Tbc1d14 3′-UTR mutation. STABLE 5: the qRT-PCR results were not consistent with that of array-based screening. STABLE 6: the primers designed for qRT-PCR validation of four autophagy associated genes.
