# Supplementary material for: miR-146b-5p Plays a Critical Role in the Regulation of Autophagy in ∆per Brucella melitensis-Infected RAW264.7 Cells
Source: Biomed Res Int. 2020 Jan 19;2020:1953242. doi: 10.1155/2020/1953242 (PMC6995328; doi:10.1155/2020/1953242)
Supplement: Supplementary Materials — Supplementary Figure 1: the technology roadmap: miRNA and mRNA profiling of macrophages infected with B. melitensis M5-90 ∆per was performed and differentially expressed miRNAs and mRNA were determined. Among the deregulated miRNA-mRNA networks, inhibitory effects of miR-146b-5p on autophagy were observed and the mechanism was analyzed. Supplementary Figure 2: construction and characterization of the per deletion mutant of B. melitensis M5-90: Kana gene fragment replaced the coding region of per gene in B. melitensis M5-90 using homologous recombination to construct deleted strains of B. melitensis M5-90 ∆per. Supplementary Figure 3: validation for the relative expression of Slc5a3 of RAW264.7 cell transfected with miR-146a-5p mimic and miR-NC mimic by qRT-PCR. Supplementary Figure 4: validation for the relative expression of Slc5a3 of RAW264.7 cell transfected with miR-146b-5p mimic and miR-NC mimic by qRT-PCR. STABLE 1: the primers designed for constructing the Per deletion mutant of B. melitensis. STABLE 2: the primers designed for qRT-PCR validation of target gene. STABLE 3: the primers designed for cloning of Tbc1d14 3′-UTR and GAPDH3′-UTR. STABLE 4: the primers designed for Tbc1d14 3′-UTR mutation. STABLE 5: the qRT-PCR results were not consistent with that of array-based screening. STABLE 6: the primers designed for qRT-PCR validation of four autophagy associated genes. [file 1953242.f1.doc]

**Supplementary Figure 1**

The technology roadmap: miRNA and mRNA profiling of macrophages infected with *B. melitensis* M5-90 ∆per were performed, and differentially expressed miRNAs and mRNA were determined. Among the deregulated miRNA-mRNA networks, inhibitory effects of miR-146b-5p on autophagy were observed and the mechanism was analyzed.

**Supplementary Figure 2**

Construction and characterization of the *per* deletion mutant of *B. melitensis* M5-90: Kana gene fragment replaced the coding region of *per* gene in *B. melitensis* M5-90. Using homologous recombination to construct deleted strains of *B. melitensis* M5-90 ∆per.

**Supplementary Figure 3**

Validation the relative expression of Slc5a3 of RAW264.7 cell transfected with miR-146a-5p mimic and miR-NC mimic by qRT-PCR

**Supplementary Figure 4**

Validation the relative expression of Slc5a3 of RAW264.7 cell transfected with miR-146b-5p mimic and miR-NC mimic by qRT-PCR

**S. 1.**

**Constructing *B. melitensis* M5-90 ∆per**

**Infection of RAW264.7 with *B.melitensis* or *B. melitensis* M5-90 ∆per**

**miRNA microarray analyses**

**The validated differentially expressed miRNAs**

**The putative targets**

**mRNA array analyses**

**The validated differentially expressed targets**

**miRNA-mRNA interaction analyses**

**miRNA-mRNA interaction mechanism model**

**S. 2.**


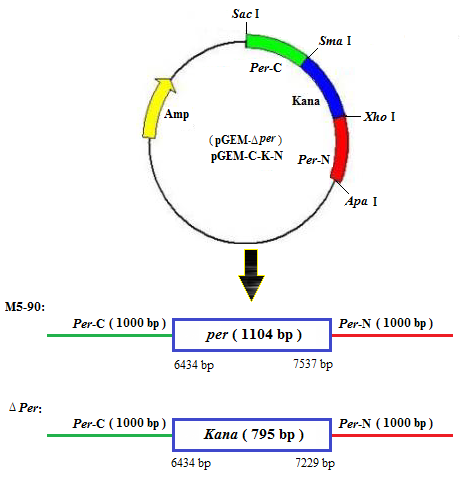


1. **3.**


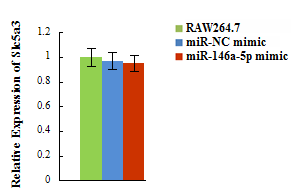


**S. 4.**


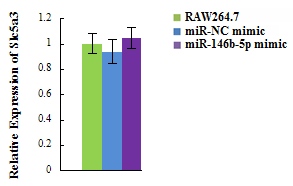


STABLE 1: The primers designed for constructing the *Per* deletion mutant of B. melitensis

| Primer | Sequence (5’-3’) | Inserted Enzyme |
| --- | --- | --- |
| *per*-N-F | GCGCGGGCCCAAGCGTCGCACCTCGCAA | Apa I |
| *per*-N-R | CGCGCTCGAGGTATGGATCATTCCTTAGGGGCG | Xho I |
| *Kana ^r^-*F1 | GCGCCTCGAGATGATTGAACAAGATGGATT | Xho I |
| *Kana ^r^-*R1 | CGCGCCCGGGTCAGAAGAACTCGTCAAG | Sma I |
| *per*-C-F | GCGCCCCGGGTGAGACGATTTCGTATGATA | Sma I |
| *per*-C-R | CGCGGAGCTCCCCGTTGTGACCTATCA | Sac I |

STABLE 2: The primers designed for qRT-PCR validation of target gene

| Gene Symbol | Genbank ID | Primer Sequence (5’-3’) |
| --- | --- | --- |
| Myo1d | NM_177390.3 | F: GGCCAACCTTAGGCTCAGATT |
|  |  | R: GAAACAACCACTTCTCCGATGAA |
| Angptl2 | NM_007426.4 | F: AACGCCTTAACCCATCTCCTTT |
|  |  | R: CCCCGAGTCTGTGGATTGAC |
| Tbc1d14 | NM_001113362.1 | F: TGGGATGGAAGCTGTTTGG |
|  |  | R: TGGGTCTTCTTTGAATCTTTCTGA |
| Slc5a3 | NM_017391.3 | F: CCGTGACTCTTACGGGTGTCA |
|  |  | R: TGATGTAGGAATTGAAGGATTCATAGC |
| Zbtb8a | NM_028603.4 | F: CACCACAGCCACGTTTCAGA |
|  |  | R: ACGAAGTCCAGGATGACTGTGA |
| Plxna2 | NM_008882.2 | F: GACTTGGATCGATCAGGAATCC |
|  |  | R: TCCTCAATGCCTGGGAACAG |
| Cbx8 | NM_013926.1 | F: GAGAAAGTGGTCGTTACAGATGTGA |
|  |  | R: TGGTCCGTGTTGCTCTCCTT |
| Zbtb42 | NM_001100460.1 | F: CCAGGTTTGGCTGGGATCTT |
|  |  | R: CGGCGCTCATTTCTAGGAAT |
| Nipal3 | NM_028995.3 | F: ACCGGGCGGTGTCTACTG |
|  |  | R: CAATGGAATAATCTCTAAGGACTGGAA |
| Psrc1 | NM_001190161.1 | F: TGCCCGGACAACAGCAA |
|  |  | R: GCTAGCTGAGGACTGTGGAACTG |

STABLE 3: The primers designed for cloning of Tbc1d14 3’-UTR and GAPDH3’-UTR

| Primer | Sequence (5’-3’) | Inserted Enzyme |
| --- | --- | --- |
| F1-Tbc1d14-3’UTR | GGCTCAGGGTAACTCACT |  |
| R1-Tbc1d14-3’UTR | AAGGGACATTCAGCTAAAGC |  |
| F2-Tbc1d14-3’UTR | CAGAGTTTGCTCCTCTTTAAC |  |
| R2-Tbc1d14-3’UTR | TAGTGCGCATCTTATGGC |  |
| F3-Tbc1d14-3’UTR | TGAAAGGCTGATGTCTGA |  |
| R3-Tbc1d14-3’UTR | TAAGTAACAATTTTACATGACT |  |
| F-Tbc1d14-3’UTR | ACGTGTTTAAACGGCTCAGGGTAACTCACT | Pme I |
| R-Tbc1d14-3’UTR | GGCCGTCGACTAAGTAACAATTTTACATGACTTTATT | Sal I |
| F-GAPDH-3’UTR | ACGTGTTTAAACGAAACCCTGGACCACCCAC | Pme I |
| R-GAPDH-3’UTR | GGCCGTCGACGTGGGTGCAGCGAACTTTATT | Sal I |

**STABLE 4: The primers designed for Tbc1d14 3’-UTR mutation**

| Primer | Sequence (5’-3’) | Inserted Enzyme |
| --- | --- | --- |
| F1-mut-Tbc1d14-3’UTR | GGCTCAGGGTAACTCACT |  |
| R1-mut-Tbc1d14-3’UTR | AAGGGACATTCAGCTAAAGC |  |
| F2-mut-Tbc1d14-3’UTR | CAGAGTTTGCTCCTCTTTAAC |  |
| R2-mut-Tbc1d14-3’UTR | GGCATGGAGTCTTGTTGACTCCAGTCATGC ATGCCCACAG |  |
| F3-mut-Tbc1d14-3’UTR | CTGTGGGCATGCATGACTGGAGTCAACAAG ACTCCATGCC |  |
| R3-mut-Tbc1d14-3’UTR | TAAGTAACAATTTTACATGACT |  |
| F-mut-Tbc1d14-3’UTR | ACGTGTTTAAACGGCTCAGGGTAACTCACT | Pme I |
| R-mut-Tbc1d14-3’UTR | GGCCGTCGACTAAGTAACAATTTTACATGACTTTATT | Sal I |

STABLE 5: The qRT-PCR results were not consistent with that of array-based screening.

|  |  | Group1  M5-90 | Group2  Per |  |  |
| --- | --- | --- | --- | --- | --- |
| Reporter Name | p-value | Mean | Mean | Log2 (G2/G1) | qRT-PCR validation ΔΔCт value |
| mmu-miR-32-5p | 3.88E-02 | 88 | 111 | 0.34 | 0.74 |
| mmu-miR-21a-5p | 1.91E-02 | 3,465 | 6,380 | 0.88 | 0.72 |
| mmu-miR-7221-3p | 4.37E-02 | 48 | 27 | -0.81 | 0.22 |

STABLE 6: The primers designed for qRT-PCR validation of four autophagy associated genes.

| Gene Symbol | Primer Sequence (5’-3’) |
| --- | --- |
| Iigp1 | F: GAGCCTGTAGCAGTGAAGGT |
|  | R: GCTGACCCATGACTTCAAGC |
| Nrbp2 | F: CCTGTCTTTCTCTCTGCGTT |
|  | R: TTCGGAGTTCTTCCCGTTCAG |
| Trp53inp1 | F: AAGTGGTCCCAGAATGGAAGC |
|  | R: CTGGGAAGGGCGAAAACTCT |
| Irgm1 | F: AGGAAGAAAGGGGTGACGTTC |
|  | R: CACAGGTCCTCTGTCTGTGAA |
